# *N*,*N*′-Bis(4-chloro­phen­yl)maleamide

**DOI:** 10.1107/S1600536811017636

**Published:** 2011-05-14

**Authors:** K. Shakuntala, Sabine Foro, B. Thimme Gowda

**Affiliations:** a Department of Chemistry, Mangalore University, Mangalagangotri 574 199, Mangalore, India; b Institute of Materials Science, Darmstadt University of Technology, Petersenstrasse 23, D-64287 Darmstadt, Germany

## Abstract

In the crystal of the title compound, C_16_H_12_Cl_2_N_2_O_2_, the two C=O groups are *anti* to each other, while one of them is *syn* and the other is *anti* to their adjacent C—H bonds. The two benzene rings are oriented at an inter­planar angle of 56.4 (1)°, while the dihedral angles between the central amide group (N–C–C–C–C–N) and these rings are 3.6 (1) and 54.1 (1)°. An intra­molecular N—H⋯O hydrogen bond occurs. In the crystal, inter­molecular N—H⋯O hydrogen bonds link the mol­ecules into infinite chains along the *a* axis.

## Related literature

For our study of the effect of substituents on the structures of *N*-(ar­yl)-amides, see: Gowda *et al.* (2004 ▶, 2011 ▶) and on the structures of *N*-(ar­yl)-methane­sulfonamides, see: Gowda *et al.* (2007 ▶).
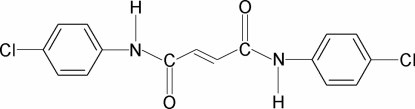


## Experimental

### 

#### Crystal data

C_16_H_12_Cl_2_N_2_O_2_
*M* *_r_* = 335.18
Monoclinic, 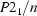

*a* = 9.2397 (7) Å
*b* = 13.0154 (8) Å
*c* = 13.1239 (9) Å
β = 107.916 (9)°
*V* = 1501.73 (18) Å^3^
*Z* = 4
Mo *K*α radiation
μ = 0.44 mm^−1^
*T* = 293 K
0.44 × 0.44 × 0.32 mm

#### Data collection

Oxford Diffraction Xcalibur diffractometer with a Sapphire CCD detector
Absorption correction: multi-scan (*CrysAlis RED*; Oxford Diffraction, 2009 ▶) *T*_min_ = 0.830, *T*_max_ = 0.872
6063 measured reflections
3065 independent reflections
2523 reflections with *I* > 2σ(*I*)
*R*_int_ = 0.011

#### Refinement

*R*[*F* ^2^ > 2σ(*F* ^2^)] = 0.033
*wR*(*F* ^2^) = 0.091
*S* = 1.06
3065 reflections
205 parameters
2 restraints
H atoms treated by a mixture of independent and constrained refinement
Δρ_max_ = 0.22 e Å^−3^
Δρ_min_ = −0.24 e Å^−3^

### 

Data collection: *CrysAlis CCD* (Oxford Diffraction, 2009 ▶); cell refinement: *CrysAlis RED* (Oxford Diffraction, 2009 ▶); data reduction: *CrysAlis RED*; program(s) used to solve structure: *SHELXS97* (Sheldrick, 2008 ▶); program(s) used to refine structure: *SHELXL97* (Sheldrick, 2008 ▶); molecular graphics: *PLATON* (Spek, 2009 ▶); software used to prepare material for publication: *SHELXL97*.

## Supplementary Material

Crystal structure: contains datablocks I, global. DOI: 10.1107/S1600536811017636/bq2300sup1.cif

Structure factors: contains datablocks I. DOI: 10.1107/S1600536811017636/bq2300Isup2.hkl

Supplementary material file. DOI: 10.1107/S1600536811017636/bq2300Isup3.cml

Additional supplementary materials:  crystallographic information; 3D view; checkCIF report

## Figures and Tables

**Table 1:** Hydrogen-bond geometry (Å, °)

*D*—H⋯*A*	*D*—H	H⋯*A*	*D*⋯*A*	*D*—H⋯*A*
N1—H1*N*⋯O2^i^	0.84 (1)	2.05 (2)	2.8836 (16)	169 (2)
N2—H2*N*⋯O1	0.86 (1)	1.83 (2)	2.6639 (17)	162 (2)

Symmetry code: (i) 
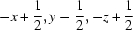
.

**Fig. 1.**
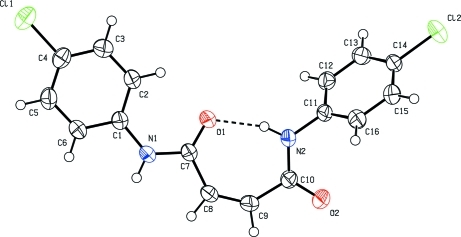
Molecular structure of (I), showing the atom labelling scheme and displacement ellipsoids are drawn at the 50% probability level. Hydrogen bonding is shown as dashed line.

**Fig. 2.**
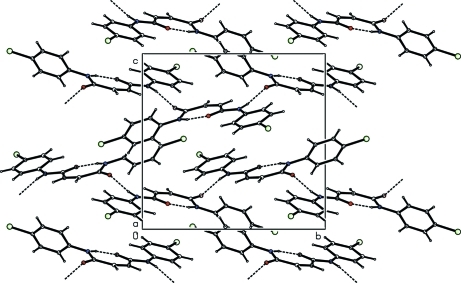
Molecular packing of (I) with hydrogen bonding shown as dashed lines.

## supplementary crystallographic information

### Comment

The amide moiety is an important constituent of many biologically significant compounds. As a part of studying the effect of substitutions on the structures of this class of compounds (Gowda *et al.*, 2004, 2007, 2011), the crystal structure of *N*,*N*-bis(4-chlorophenyl)-maleamide has been determined (I) (Fig. 1). In the structure, the conformations of N—H and C=O bonds in both the amide groups of the C—NH—CO—CH ═CH—CO—NH—C segment are *anti* to each other. The two C=O bonds are also *anti* to each other, while one of them is *syn* to the adjacent C—H bond and the other is *anti* to its adjacent C—H bond, similar to that observed in *N*,*N*-bis(phenyl)-maleamide (II) (Gowda *et al.*, 2011).

Further, C1—N1—C7—C8 and C11—N2—C10—C9 segments are nearly linear. The torsion angles of C2—C1—N1—C7 and C6—C1—N1—C7 are -7.0 (3)° and 174.2 (2)°, respectively, compared to the values of 174.4 (3)° and -4.9 (4)° in (II). The torsion angles of C12—C11—N2—C10 and C16—C11—N2—C10 are 122.7 (2)° and -59.1 (2)°, in contrast to the values of 40.4 (4)° and -143.9 (3)° in (II).

The two phenyl rings in (I) make an interplanar angle of 56.4 (1)°, compared to the value of 41.2 (1)° in (II). The two benzene rings (C1 to C6 and C11 to C16) make the dihedral angles of 3.6 (1)° and 54.1 (1)°, respectively, with the central amide group (N1—C7—C8—C9—C10—N2), compared to the corresponding values of 8.0 (1)° and 38.3 (1)° in (II).

The crystal structure exhibits both the intramolecular and intermolecular N–H···O hydrogen bonding (Table 1). The packing of molecules through intermolecular N–H···O hydrogen bonds is shown in Fig. 2.

### Experimental

A mixture of maleic acid (0.2 mol) and phosphorous oxy chloride (0.3 mol) were refluxed for 3 hrs on a water bath at 95° C. 4-Chloroaniline was added dropwise with stirring and continuing heating for about 30 min. It was later kept aside for 12 hrs for completion of the reaction. The reaction mixture was then added to ice. The precipitated product was washed with water, dilute HCl, dilute NaOH and again with water. The product was filtered, dried and recrystallized from DMF.

Prism like dark-grey single crystals of the title compound used in X-ray diffraction studies were obtained by a slow evaporation of its DMF solution at room temperature.

### Refinement

The H atoms of the NH groups were located in a difference map and later restrained to the distance N—H = 0.86 (2) Å. The other H atoms were positioned with idealized geometry using a riding model with C—H = 0.93 Å. All H atoms were refined with isotropic displacement parameters (set to 1.2 times of the *U*_eq_ of the parent atom).

### Crystal data

**Table:**

C_16_H_12_Cl_2_N_2_O_2_	*F*(000) = 688
*M**_r_* = 335.18	*D*_x_ = 1.482 Mg m^−^^3^
Monoclinic, *P*2_1_/*n*	Mo *K*α radiation, λ = 0.71073 Å
Hall symbol: -P 2yn	Cell parameters from 3188 reflections
*a* = 9.2397 (7) Å	θ = 2.8–27.9°
*b* = 13.0154 (8) Å	µ = 0.44 mm^−^^1^
*c* = 13.1239 (9) Å	*T* = 293 K
β = 107.916 (9)°	Prism, dark grey
*V* = 1501.73 (18) Å^3^	0.44 × 0.44 × 0.32 mm
*Z* = 4	

### Data collection

**Table:**

Oxford Diffraction Xcalibur diffractometer with a Sapphire CCD detector	3065 independent reflections
Radiation source: fine-focus sealed tube	2523 reflections with *I* > 2σ(*I*)
graphite	*R*_int_ = 0.011
Rotation method data acquisition using ω scans	θ_max_ = 26.4°, θ_min_ = 2.8°
Absorption correction: multi-scan (*CrysAlis RED*; Oxford Diffraction, 2009)	*h* = −11→10
*T*_min_ = 0.830, *T*_max_ = 0.872	*k* = −14→16
6063 measured reflections	*l* = −16→11

### Refinement

**Table:**

Refinement on *F*^2^	Primary atom site location: structure-invariant direct methods
Least-squares matrix: full	Secondary atom site location: difference Fourier map
*R*[*F*^2^ > 2σ(*F*^2^)] = 0.033	Hydrogen site location: inferred from neighbouring sites
*wR*(*F*^2^) = 0.091	H atoms treated by a mixture of independent and constrained refinement
*S* = 1.06	*w* = 1/[σ^2^(*F*_o_^2^) + (0.0494*P*)^2^ + 0.3163*P*] where *P* = (*F*_o_^2^ + 2*F*_c_^2^)/3
3065 reflections	(Δ/σ)_max_ = 0.001
205 parameters	Δρ_max_ = 0.22 e Å^−^^3^
2 restraints	Δρ_min_ = −0.24 e Å^−^^3^

### Special details

**Table:**

Experimental. CrysAlis RED (Oxford Diffraction, 2009) Empirical absorption correction using spherical harmonics, implemented in SCALE3 ABSPACK scaling algorithm.
Geometry. All e.s.d.'s (except the e.s.d. in the dihedral angle between two l.s. planes) are estimated using the full covariance matrix. The cell e.s.d.'s are taken into account individually in the estimation of e.s.d.'s in distances, angles and torsion angles; correlations between e.s.d.'s in cell parameters are only used when they are defined by crystal symmetry. An approximate (isotropic) treatment of cell e.s.d.'s is used for estimating e.s.d.'s involving l.s. planes.
Refinement. Refinement of *F*^2^ against ALL reflections. The weighted *R*-factor *wR* and goodness of fit *S* are based on *F*^2^, conventional *R*-factors *R* are based on *F*, with *F* set to zero for negative *F*^2^. The threshold expression of *F*^2^ > σ(*F*^2^) is used only for calculating *R*-factors(gt) *etc*. and is not relevant to the choice of reflections for refinement. *R*-factors based on *F*^2^ are statistically about twice as large as those based on *F*, and *R*- factors based on ALL data will be even larger.

### Fractional atomic coordinates and isotropic or equivalent isotropic displacement parameters (Å^2^)

**Table:**

	*x*	*y*	*z*	*U*_iso_*/*U*_eq_	
Cl1	1.02985 (5)	−0.18771 (4)	0.07433 (4)	0.05576 (15)	
Cl2	0.34374 (6)	0.72529 (3)	−0.01488 (4)	0.06130 (16)	
O1	0.49648 (15)	0.13865 (8)	0.14544 (12)	0.0566 (4)	
O2	0.10969 (14)	0.32142 (8)	0.19093 (9)	0.0449 (3)	
N1	0.49484 (15)	−0.03027 (10)	0.18426 (11)	0.0380 (3)	
H1N	0.4540 (19)	−0.0748 (13)	0.2131 (13)	0.046*	
N2	0.30826 (16)	0.29745 (10)	0.12704 (11)	0.0393 (3)	
H2N	0.3780 (18)	0.2545 (14)	0.1250 (14)	0.047*	
C1	0.62405 (17)	−0.06341 (11)	0.15703 (12)	0.0349 (3)	
C2	0.7035 (2)	−0.00167 (13)	0.10578 (14)	0.0479 (4)	
H2	0.6734	0.0660	0.0888	0.057*	
C3	0.8268 (2)	−0.04074 (14)	0.08021 (14)	0.0479 (4)	
H3	0.8799	0.0007	0.0462	0.057*	
C4	0.87128 (18)	−0.14080 (13)	0.10489 (12)	0.0399 (4)	
C5	0.79395 (19)	−0.20323 (12)	0.15518 (13)	0.0430 (4)	
H5	0.8240	−0.2711	0.1711	0.052*	
C6	0.67118 (18)	−0.16405 (11)	0.18174 (13)	0.0391 (3)	
H6	0.6196	−0.2057	0.2166	0.047*	
C7	0.43672 (17)	0.06548 (11)	0.17594 (12)	0.0367 (3)	
C8	0.29546 (19)	0.07352 (12)	0.20689 (13)	0.0410 (4)	
H8	0.2649	0.0130	0.2319	0.049*	
C9	0.20588 (19)	0.15430 (12)	0.20424 (13)	0.0423 (4)	
H9	0.1231	0.1380	0.2274	0.051*	
C10	0.20609 (17)	0.26486 (11)	0.17309 (11)	0.0346 (3)	
C11	0.31503 (17)	0.40084 (11)	0.09296 (12)	0.0346 (3)	
C12	0.44804 (17)	0.45625 (12)	0.13303 (12)	0.0369 (3)	
H12	0.5318	0.4261	0.1825	0.044*	
C13	0.45728 (18)	0.55621 (12)	0.10004 (12)	0.0386 (3)	
H13	0.5469	0.5935	0.1267	0.046*	
C14	0.33194 (18)	0.59989 (11)	0.02710 (12)	0.0371 (3)	
C15	0.19922 (18)	0.54565 (13)	−0.01497 (13)	0.0432 (4)	
H15	0.1160	0.5759	−0.0649	0.052*	
C16	0.19120 (19)	0.44518 (12)	0.01807 (13)	0.0421 (4)	
H16	0.1024	0.4075	−0.0102	0.051*	

### Atomic displacement parameters (Å^2^)

**Table:**

	*U*^11^	*U*^22^	*U*^33^	*U*^12^	*U*^13^	*U*^23^
Cl1	0.0450 (2)	0.0601 (3)	0.0653 (3)	0.0114 (2)	0.0217 (2)	−0.0056 (2)
Cl2	0.0756 (3)	0.0331 (2)	0.0800 (3)	0.0011 (2)	0.0311 (3)	0.0178 (2)
O1	0.0608 (8)	0.0259 (6)	0.1020 (10)	0.0056 (5)	0.0528 (7)	0.0123 (6)
O2	0.0515 (7)	0.0384 (6)	0.0541 (7)	0.0094 (5)	0.0300 (5)	0.0004 (5)
N1	0.0443 (7)	0.0262 (6)	0.0500 (7)	0.0015 (5)	0.0242 (6)	0.0079 (5)
N2	0.0467 (8)	0.0271 (6)	0.0530 (8)	0.0088 (6)	0.0282 (6)	0.0076 (6)
C1	0.0381 (8)	0.0284 (7)	0.0394 (8)	0.0016 (6)	0.0139 (6)	0.0004 (6)
C2	0.0580 (10)	0.0334 (8)	0.0624 (10)	0.0103 (8)	0.0336 (9)	0.0138 (8)
C3	0.0537 (10)	0.0436 (9)	0.0549 (10)	0.0034 (8)	0.0293 (8)	0.0084 (8)
C4	0.0377 (8)	0.0421 (9)	0.0393 (8)	0.0044 (7)	0.0108 (6)	−0.0068 (7)
C5	0.0449 (9)	0.0310 (8)	0.0511 (9)	0.0061 (7)	0.0116 (7)	−0.0008 (7)
C6	0.0435 (8)	0.0282 (7)	0.0463 (8)	−0.0024 (6)	0.0147 (7)	0.0022 (6)
C7	0.0425 (8)	0.0262 (7)	0.0460 (8)	0.0014 (6)	0.0206 (7)	0.0033 (6)
C8	0.0500 (9)	0.0293 (7)	0.0523 (9)	0.0001 (7)	0.0285 (8)	0.0075 (7)
C9	0.0490 (9)	0.0373 (8)	0.0515 (9)	0.0015 (7)	0.0315 (8)	0.0064 (7)
C10	0.0415 (8)	0.0313 (7)	0.0353 (7)	0.0034 (6)	0.0180 (6)	−0.0005 (6)
C11	0.0433 (8)	0.0271 (7)	0.0403 (8)	0.0060 (6)	0.0231 (6)	0.0030 (6)
C12	0.0389 (8)	0.0381 (8)	0.0364 (8)	0.0076 (7)	0.0154 (6)	0.0060 (6)
C13	0.0417 (8)	0.0358 (8)	0.0421 (8)	−0.0028 (7)	0.0183 (7)	−0.0009 (6)
C14	0.0489 (9)	0.0269 (7)	0.0430 (8)	0.0043 (6)	0.0252 (7)	0.0052 (6)
C15	0.0414 (8)	0.0396 (9)	0.0486 (9)	0.0087 (7)	0.0140 (7)	0.0113 (7)
C16	0.0386 (8)	0.0350 (8)	0.0525 (9)	−0.0001 (7)	0.0136 (7)	0.0040 (7)

### Geometric parameters (Å, °)

**Table:**

Cl1—C4	1.7440 (16)	C5—H5	0.9300
Cl2—C14	1.7367 (15)	C6—H6	0.9300
O1—C7	1.2280 (18)	C7—C8	1.485 (2)
O2—C10	1.2322 (18)	C8—C9	1.332 (2)
N1—C7	1.3481 (19)	C8—H8	0.9300
N1—C1	1.4149 (19)	C9—C10	1.496 (2)
N1—H1N	0.843 (14)	C9—H9	0.9300
N2—C10	1.3369 (19)	C11—C12	1.382 (2)
N2—C11	1.4255 (19)	C11—C16	1.384 (2)
N2—H2N	0.860 (14)	C12—C13	1.382 (2)
C1—C6	1.387 (2)	C12—H12	0.9300
C1—C2	1.393 (2)	C13—C14	1.378 (2)
C2—C3	1.380 (2)	C13—H13	0.9300
C2—H2	0.9300	C14—C15	1.375 (2)
C3—C4	1.374 (2)	C15—C16	1.387 (2)
C3—H3	0.9300	C15—H15	0.9300
C4—C5	1.377 (2)	C16—H16	0.9300
C5—C6	1.383 (2)		
			
C7—N1—C1	127.41 (13)	C9—C8—C7	129.78 (14)
C7—N1—H1N	116.8 (13)	C9—C8—H8	115.1
C1—N1—H1N	115.7 (13)	C7—C8—H8	115.1
C10—N2—C11	123.13 (13)	C8—C9—C10	135.51 (14)
C10—N2—H2N	116.6 (13)	C8—C9—H9	112.2
C11—N2—H2N	119.9 (13)	C10—C9—H9	112.2
C6—C1—C2	118.92 (14)	O2—C10—N2	123.26 (14)
C6—C1—N1	117.23 (13)	O2—C10—C9	117.45 (13)
C2—C1—N1	123.84 (14)	N2—C10—C9	119.29 (13)
C3—C2—C1	120.13 (15)	C12—C11—C16	119.78 (14)
C3—C2—H2	119.9	C12—C11—N2	119.54 (14)
C1—C2—H2	119.9	C16—C11—N2	120.66 (14)
C4—C3—C2	120.08 (16)	C11—C12—C13	120.37 (14)
C4—C3—H3	120.0	C11—C12—H12	119.8
C2—C3—H3	120.0	C13—C12—H12	119.8
C3—C4—C5	120.71 (15)	C14—C13—C12	119.10 (15)
C3—C4—Cl1	119.28 (13)	C14—C13—H13	120.5
C5—C4—Cl1	120.00 (13)	C12—C13—H13	120.5
C4—C5—C6	119.34 (15)	C15—C14—C13	121.49 (14)
C4—C5—H5	120.3	C15—C14—Cl2	119.37 (12)
C6—C5—H5	120.3	C13—C14—Cl2	119.12 (12)
C5—C6—C1	120.82 (15)	C14—C15—C16	119.04 (15)
C5—C6—H6	119.6	C14—C15—H15	120.5
C1—C6—H6	119.6	C16—C15—H15	120.5
O1—C7—N1	122.38 (14)	C11—C16—C15	120.21 (15)
O1—C7—C8	123.75 (14)	C11—C16—H16	119.9
N1—C7—C8	113.87 (13)	C15—C16—H16	119.9
			
C7—N1—C1—C6	174.16 (15)	C11—N2—C10—O2	−0.6 (3)
C7—N1—C1—C2	−7.0 (3)	C11—N2—C10—C9	178.80 (14)
C6—C1—C2—C3	0.1 (3)	C8—C9—C10—O2	−173.19 (19)
N1—C1—C2—C3	−178.74 (16)	C8—C9—C10—N2	7.4 (3)
C1—C2—C3—C4	0.2 (3)	C10—N2—C11—C12	122.66 (17)
C2—C3—C4—C5	0.0 (3)	C10—N2—C11—C16	−59.1 (2)
C2—C3—C4—Cl1	−178.68 (14)	C16—C11—C12—C13	1.0 (2)
C3—C4—C5—C6	−0.6 (2)	N2—C11—C12—C13	179.29 (13)
Cl1—C4—C5—C6	178.10 (12)	C11—C12—C13—C14	0.3 (2)
C4—C5—C6—C1	0.9 (2)	C12—C13—C14—C15	−1.3 (2)
C2—C1—C6—C5	−0.7 (2)	C12—C13—C14—Cl2	−179.70 (11)
N1—C1—C6—C5	178.25 (14)	C13—C14—C15—C16	0.9 (2)
C1—N1—C7—O1	−2.6 (3)	Cl2—C14—C15—C16	179.31 (12)
C1—N1—C7—C8	177.38 (14)	C12—C11—C16—C15	−1.4 (2)
O1—C7—C8—C9	3.0 (3)	N2—C11—C16—C15	−179.66 (14)
N1—C7—C8—C9	−176.98 (17)	C14—C15—C16—C11	0.5 (2)
C7—C8—C9—C10	−0.8 (3)		

### Hydrogen-bond geometry (Å, °)

**Table:**

*D*—H···*A*	*D*—H	H···*A*	*D*···*A*	*D*—H···*A*
N1—H1N···O2^i^	0.84 (1)	2.05 (2)	2.8836 (16)	169.(2)
N2—H2N···O1	0.86 (1)	1.83 (2)	2.6639 (17)	162.(2)

Symmetry codes: (i) −*x*+1/2, *y*−1/2, −*z*+1/2.

## References

[bb1] Gowda, B. T., Foro, S. & Fuess, H. (2007). *Acta Cryst.* E**63**, o2597.

[bb2] Gowda, B. T., Foro, S., Shakuntala, K. & Fuess, H. (2011). *Acta Cryst.* E**67**, o117. 10.1107/S1600536810051330 PMC3050154 21522628

[bb3] Gowda, B. T., Svoboda, I. & Fuess, H. (2004). *Z. Naturforsch. Teil A*, **55**, 845–852.

[bb4] Oxford Diffraction (2009). *CrysAlis CCD* and *CrysAlis RED* Oxford Diffraction Ltd, Yarnton, England.

[bb5] Sheldrick, G. M. (2008). *Acta Cryst.* A**64**, 112–122. 10.1107/S0108767307043930 18156677

[bb6] Spek, A. L. (2009). *Acta Cryst.* D**65**, 148–155. 10.1107/S090744490804362X PMC2631630 19171970

